# Initial Experience of a Series of Single-Port Robotic Pancreatoduodenectomy Using the Da Vinci SP System

**DOI:** 10.7759/cureus.94403

**Published:** 2025-10-12

**Authors:** Nim Choi, Chi Ian Kuok, Antonio Fernandes Das Neves, Chin Wan Leong, Ka Man Ng

**Affiliations:** 1 General Surgery, Conde S. Januário Hospital, Macau, MAC

**Keywords:** laparoscopic pancreaticoduodenectomy (lpd), minimally invasive pancreaticoduodenectomy (mipd), minimally invasive surgery, multiport robotic pancreaticoduodenectomy (mprpd), pancreatic cancer, pancreatic surgery, robotic pancreatoduodenectomy, single-port robotic-assisted pancreatoduodenectomy (sprpd), whipple procedure

## Abstract

Objective: This study reports the initial series of patients who underwent single-port robotic-assisted pancreatoduodenectomy (SPRPD) using the da Vinci SP system (Intuitive Surgical, Inc., Sunnyvale, CA), demonstrating its technical feasibility and short-term perioperative outcomes. We also aimed to provide a standardized step-by-step SPRPD procedure to facilitate the learning curve.

Methods: We analyzed clinical data, surgical steps, and postoperative recovery of patients who underwent SPRPD between August 2024 and December 2024. Short-term outcomes were assessed.

Results: Seven patients underwent SPRPD using the da Vinci SP system. Fourteen operative steps were summarized as key elements of the SPRPD procedure. The average age was 68 years, with five males and two females. Diagnoses included pancreatic head adenocarcinoma (three cases), duodenal adenocarcinoma (two cases), common bile duct adenocarcinoma (one case), and intraductal papillary mucinous neoplasm (IPMN, low grade) (one case). The average single-arm docking time was 3.5 minutes. The mean operative time was 584 minutes (9.7 hours), with an estimated blood loss of 143 ml; only one patient required a blood transfusion. No patient required conversion to laparoscopic or open surgery. Regarding short-term oncological outcomes, the largest tumor measured 2.7 cm, and the average number of harvested lymph nodes was 12. A clear resection margin (R0) was achieved in six patients (85.7%), whereas one patient with pancreatic head cancer had a positive superior mesenteric vein margin (14.3%). Delicate surgical anatomical dissection and all types of pancreato-biliary-enteral reconstruction were successfully performed. Postoperative complications included clinically relevant postoperative pancreatic fistula (CR-POPF, grade B) in one patient (14.3%) and delayed gastric emptying in two patients (28.6%). Major complications (Clavien-Dindo grade ≥ III) occurred in four patients (57.1%). There was no 30- or 90-day mortality. Patients resumed oral intake after a mean of 9.2 days, with an average postoperative hospital stay of 22.2 days. Two patients (28.6%) were readmitted because of delayed gastric emptying and vomiting.

Conclusions: SPRPD is technically feasible with favorable short-term outcomes. This approach facilitates precise anatomical dissection with negative margins and allows the execution of all types of anastomoses. However, the adoption of SPRPD as a standard approach remains controversial. Long-term outcomes and randomized controlled trials are necessary to define the role of SPRPD more clearly. Standardized operative techniques may shorten the learning curve.

## Introduction

Pancreatoduodenectomy (PD), commonly known as the “Whipple's procedure,” is the only radical treatment and potential curative option for pancreatic head cancers. PD was first performed by Codivilla in 1898 [1] and was later popularized by Allen Oldfather Whipple, who reported the first successful one-stage PD in 1941 [2].

Minimally invasive pancreaticoduodenectomy (MIPD), specifically laparoscopic pancreatoduodenectomy (LPD), was first reported by Gagner and Pomp in 1994, offering improvements over the traditional open approach [3]. However, LPD remains limited by its technical complexity and the advanced laparoscopic skills required for pancreatic reconstruction, which prevents its widespread adoption.

In recent years, robotic surgical systems have been introduced to overcome these limitations. The first multi-port robotic pancreaticoduodenectomy (MPRPD) was performed by P. C. Giulianotti in 2001 and published in 2003 [4]. Since then, robot-assisted pancreaticoduodenectomy (RPD) has gained wide acceptance and demonstrated safety and feasibility comparable to open approaches. Moreover, MIPD is now included as a recommended option in the latest National Comprehensive Cancer Network (NCCN) guidelines [5].

Advancements in robotic surgical technology have led to the development of single-port (SP) robotic systems, which are increasingly utilized in clinical practice. These systems offer several advantages, including multi-jointed wristed instruments and a fully wristed three-dimensional (3D) high-definition camera integrated within a single 25-mm shaft. They are capable of performing complex operations in hepato-biliary-pancreatic (HBP), gastrointestinal (GI), and urological surgery [6-10]. Single-port robotic-assisted pancreatoduodenectomy (SPRPD), as a new operating platform, has been rarely reported due to its inherent complexity, which limits broader clinical application and research.

This study aimed to present a series of seven consecutive SPRPD cases and to conduct a retrospective analysis assessing their perioperative outcomes at a single center, providing insights into the clinical feasibility and safety of SPRPD. By sharing the initial experience of SPRPD, we also aimed to present a standardized 14-step operative technique to assist surgeons considering the adoption of this novel surgical platform and to facilitate the learning curve.

## Materials and methods

From August 2024 to December 2024, seven patients underwent PD using the da Vinci SP system (Intuitive Surgical, Inc., Sunnyvale, CA). Diagnoses included pancreatic head adenocarcinoma (three cases), duodenal adenocarcinoma (two cases), common bile duct (CBD) adenocarcinoma (one case), and intraductal papillary mucinous neoplasm (IPMN, low grade) (one case). The average patient age was 68 years (range: 59-73 years). Diagnoses were established using endoscopic retrograde cholangiopancreatography (ERCP), computed tomography (CT), serum tumor markers, and biopsy confirmation. Imaging evaluation was performed using CT and magnetic resonance imaging (MRI) to determine disease stage. Surgical technique steps and short-term results were analyzed.

Definition of robot docking time

Docking time refers to the interval from the initiation of robotic arm setup to the moment the instruments are fully inserted and under the surgeon's control. This period included positioning the robotic arms, attaching them to the trocars (i.e., surgical ports), and inserting instruments into the patient.

Operative technique of SPRPD

Trocar Positioning and Docking

A paraumbilical incision approximately 5 cm in length was made to introduce the access port, followed by creation of pneumoperitoneum with an open technique.

Stepwise Operative Technique

The SPRPD was standardized into 14 steps (Table 1). There are three main working abdominal quadrants for the SP systems (main shaft with camera and instruments): (1) middle quadrant, (2) right upper quadrant, and (3) left upper quadrant. The lower margin is defined by the mesocolon and ligament of Treitz.

**Table 1:** Stepwise surgical technique of SPRPD standardized in 14 steps. SPRPD: single-port robotic-assisted pancreatoduodenectomy.

Procedure for dissection
1. Gastrocolic ligament opening and lesser omentum division: middle quadrant
2. Hepatic hilum exploration and gastroduodenal artery division: middle quadrant
3. Right gastric artery and right gastroepiploic artery division: middle quadrant
4. Distal stomach division or proximal duodenum transection: middle quadrant
5. Extended Kocher maneuver: middle quadrant
6. Pancreatic neck transection: middle quadrant
7. First jejunal loop transection: middle quadrant
8. Uncinate process dissection: middle quadrant
9. Cholecystectomy: right upper quadrant
10. Common bile duct transection: right upper quadrant
Procedure for reconstruction
11. Pancreatogastro or jejunostomy: left upper quadrant
12. Hepaticojejunostomy (biliary reconstruction): right upper quadrant
13. Pylorus-preserving or gastrojejunostomy (duodenojejunal reconstruction): left upper quadrant
14. Specimen extraction and closure

Procedure for Dissection

The working shaft was first moved to the middle quadrant to perform the following steps: (1) gastrocolic ligament opening and division of the lesser omentum using fenestrated bipolar and Cadiere forceps, extended to the short gastric vessels. (2) Hepatic hilum exploration and portal dissection, including division of the gastroduodenal artery and removal of hepatoduodenal ligament lymphatic tissue (group No. 12) and along the common hepatic artery (group No. 8). (3) Right gastric artery and right gastroepiploic artery division. In pylorus-preserving pancreaticoduodenectomy (PPPD), the right gastroepiploic arcade and proximal duodenal arcade were preserved to ensure adequate blood supply to the pylorus. (4) Distal stomach division or transection of the proximal duodenum approximately 3 cm distal to the pylorus using Signia. (5) An extended Kocher maneuver was performed on the left lateral edge of the aorta, and all fibrofatty and lymphatic tissue over the medial aspect of the inferior vena cava (IVC) was removed. (6) Pancreatic neck transection anterior to the superior mesenteric vein (SMV) and medial to the SMV−splenic vein junction using Signia. (7) Transection of the first jejunal loop 10 cm distal to the ligament of Treitz, followed by division of the duodenojejunal flexure and right-sided derotation of the duodenum. (8) Uncinate process dissection en bloc with all soft tissue on the right aspect of the proximal superior mesenteric artery (SMA). Subsequently, the working shaft was moved to the right upper quadrant for the following procedures: (9) cholecystectomy and (10) common hepatic duct transection above the cystic duct were performed using Signia. Bioglue was applied to the SMA dissection region, and Tisseel glue was applied to the portal vein, SMV region, and retroperitoneal space.

Procedure for Reconstruction

The reconstructive phase may vary depending on the following factors: (1) preservation of the pylorus, (2) pancreatic duct size, (3) pancreatic parenchyma texture, and (4) bile duct diameter. The primary determinants for selecting the type of pancreatic anastomosis were pancreatic tissue texture and duct diameter. Soft or fragile pancreatic tissue, or a small duct (<3 mm), generally favors transgastric pancreaticogastrostomy, whereas firm or fibrotic pancreas with a duct >4 mm typically warrants pancreatojejunostomy. In cases where the pancreatic tissue was moderate to firm and the duct was ≥3 mm, pancreatojejunostomy with the modified Blumgart technique was performed. The working shaft was then moved to the left upper quadrant for the following procedures: (11) pancreatogastro/pancreatojejunostomy. (1.1) Pancreatojejunostomy (retromesenteric route) with end-to-side duct mucosa reconstruction is preferred when the pancreatic duct is ≥3 mm. For ducts ≥5 mm, a small stent can be placed and secured with 5/0 polydioxanone (PDS) suture (total three cases: one IPMN and two pancreatic head cancers) using a two-layer end-to-side duct-to-mucosa technique. (1.2) Transgastric pancreatogastrostomy (PG) is preferred for patients with high-risk pancreatic tissue. The pancreatic stump is mobilized for at least 5 cm. Evidence suggests that pancreatic fistulas following PG are associated with reduced severity and morbidity because pancreatic juice is inactivated within the gastric lumen. However, this technique carries a higher risk of postoperative bleeding and long-term pancreatic atrophy, which may impair both endocrine and exocrine function (a total of three cases: one CBD cancer, one duodenal cancer, and one pancreatic head cancer). (1.3) Pancreatojejunostomy with the modified Blumgart technique was performed in one case (single incision laparoscopic surgery) for duodenal cancer. Subsequently, the working shaft was moved to the right upper quadrant for the following procedures: (12) hepaticojejunostomy (end-to-side) was performed 10 cm distal to the pancreaticojejunostomy (PJ) using V-Loc 4/0. The working shaft was then moved to the left upper quadrant for the following procedures: (13) duodeno/gastrojejunostomy (duodenojejunal reconstruction). (1.1) Duodenojejunostomy was performed 40 cm distal to the hepaticojejunostomy (HJ). Indocyanine green (ICG) was used, when indicated, to assess the adequacy of perfusion to the duodenal margins before performing the anastomosis (two cases of retrocolic PPPD, one CBD cancer, and one pancreatic head cancer. (1.2) Gastrojejunostomy (GJ) was performed 40 cm distal to the HJ (a total of four cases: antecolic: one pancreatic head cancer, two duodenal cancer). (14) Specimen extraction and closure: specimen extraction was completed, and two drains were placed: one at the pancreatic anastomosis site and one near the biliary anastomosis.

## Results

Patient selection and patient demographics are summarized in Table 2.

**Table 2:** Patient demographics. ASA: American Society of Anesthesiologists; SP: single port; IPMN: intraductal papillary mucinous neoplasm.

Parameter	Data
Patient number	7
Male: female	5:2
Age (years)	68 (range, 59−73)
BMI (kg/m^2^)	19.1 (range, 16.7−20.4)
ASA classification	
I	0
II	0
III	7
Tumor types	
IPMN	1
Common bile duct adenocarcinoma	1
Pancreatic head adenocarcinoma	3
Duodenal adenocarcinoma	2
Neoadjuvant chemoradiation	0
Pancreaticoenterology reconstruction	
Pancreatojejunostomy	3
Pancreaticojejunostomy (Blumgart)	1
Pancreatogastrostomy	3
Pylorus-preserving pancreatoduodenectomy	2

From August 2024 to December 2024, all patients who were diagnosed with pancreatic head adenocarcinoma, duodenal adenocarcinoma, CBD adenocarcinoma, and IPMN without any vessel involvement or distal metastasis were included in this study. Exclusion criteria included generally metastatic or unresectable disease, severe comorbidities, critical organ dysfunction, or advanced age-related frailty. Seven patients underwent PD using the da Vinci SP system. Diagnoses included pancreatic head adenocarcinoma (three cases), duodenal adenocarcinoma (two cases), CBD adenocarcinoma (one case), and low-grade IPMN (one case). The mean patient age was 68 years (range, 59−73 years), with five males and two females. The mean body mass index (BMI) was 19.1 kg/m² (range, 16.7−20.4). All patients were classified as American Society of Anesthesiologists (ASA) Physical Status III. No patient received neoadjuvant chemotherapy, and none required vascular resection at the time of SPRPD. We omitted patients who received neoadjuvant chemotherapy, and we did not consider borderline resectable cancer for induction in this study. As this is the initial series of patients who underwent SPRPD using the da Vinci SP system, we chose the easy and simple cases in this study. Maybe in future studies, as we have more experience, we will consider more complicated cases.

Intraoperative outcomes

The mean docking time was 3.5 minutes (range, three to five minutes), and the mean operative time was 584 minutes (range, 405−840 minutes). The mean estimated blood loss was 143 mL (range, 50−300 mL). One patient (14.2%) required transfusion of two units of red blood cells (RBC) (14.2%). No patient required conversion to laparoscopic or open surgery. For short-term oncological outcomes, the mean tumor size was 2.7 cm (range, 1.5−5 cm), and the mean number of harvested lymph nodes was 12 (range, 7−19). A clear resection margin was achieved in six patients (85.7%), while one patient with pancreatic head cancer had a positive SMV margin (14.3%) (Table 2). Types of anastomosis reconstruction: (a) PJ: four cases (one IPMN, two pancreatic head adenocarcinoma, and one duodenal adenocarcinoma); (b) PJ with Blumgart technique: one case (duodenal carcinoma); (c) PG: three cases, including gastrojejunostomy, in one case (duodenal adenocarcinoma) and duodenojejunostomy (DJ-PPPD) in two cases (one pancreatic head adenocarcinoma and one CBD adenocarcinoma). Operative time did not differ notably between anastomosis types: for PJ (four cases), the mean operative time was 605 minutes (range, 405−840 minutes), and for PG with or without DJ/GJ (three cases), it was 556 minutes (range, 460−640 minutes).

Postoperative outcomes

Major complications (Clavien-Dindo grade ≥ III) are shown in Table 3. Postoperative pancreatic fistula (POPF) occurred in four patients (57.1%), of which three patients (42.8%) had biochemical leaks (class A: one patient with CBD cancer, two patients with duodenal cancers) and one patient (14.3%) had a clinically relevant POPF (CR-POPF, class B: pancreatic head adenocarcinoma). No patient experienced grade C POPF, the most severe form of CR-POPF. Delayed gastric emptying occurred in two patients (28.6%) with pancreatic adenocarcinoma and duodenal adenocarcinoma. Chylous leakage (ascites) occurred in one patient (14.3%) with pancreatic adenocarcinoma and was managed conservatively. Post-pancreatectomy hemorrhage occurred in one patient (14.3%) with pancreatic head carcinoma with PG anastomosis bleeding, which was managed successfully by endoscopic hemostasis. Abdominal infection with *Enterobacter*/*Klebsiella* was noted in two patients (28.6%) with pancreatic head adenocarcinoma and duodenal adenocarcinoma. Pneumonia occurred in one patient (14.3%) with IPMN. No patient required reoperation. There were no 30-day or 90-day postoperative mortalities. Oral feeding was resumed at a mean of 9.2 days postoperatively (range, 5−17 days), and delayed oral feeding occurred in two patients due to chylous leakage (16 days) and PG hemorrhage (17 days). The median postoperative hospital stay (PHS) was 22.2 days (range, 10−40 days). Ninety-day readmission was required in two patients (28.6%) because of delayed gastric emptying and vomiting.

**Table 3:** Perioperative outcomes. EBL: estimated blood loss; CR-POPF: clinically relevant-postoperative pancreatic fistula; DGE: delayed gastric emptying; PPH: postpancreatectomy hemorrhage; PHS: postoperative hospital stay.

Parameter	Data
Operative time, minutes	584 (range, 405−840 minutes)
Docking time, minutes	3.5 (range, 3−5 minutes)
EBL, mL	143 mL (range, 50−300 mL)
Blood transfusion	1 patient (14.2%)
Conversion rate	0
CR-POPF B/C	1/7, B (14.3%)
DGE	2/7 (28.6%)
Abdominal infection	2/7 (28.6%)
PPH	1/7 (14.3%)
First oral diet, days	9.2 days (range, 5−17)
PHS, days	22.2 (range, 10−40)
Reoperation	0
90-day readmission	2/7 (28.6%)
90-day mortality	0

Postoperative pathology findings are summarized in Table 4. There were three cases of pancreatic head ductal adenocarcinoma, including (a) moderate pancreatic head adenocarcinoma, pT2N1. The nearest distance to the SMV margin was 1.5 cm; all margins were clear. Seven lymph nodes were harvested, with one positive for metastasis. (b) Ductal adenocarcinoma/IPMN with high-grade dysplasia, pT3N1M0. The SMV margin was positive. Twelve lymph nodes were harvested, with three positive for metastasis. (c) Moderately differentiated adenocarcinoma, pT3N2M0 (American Joint Committee on Cancer (AJCC), 8th edition). The closest SMV margin was 5 mm. Nineteen lymph nodes were harvested, with five positive for metastasis. There were two cases of duodenal adenocarcinoma, including (a) well-differentiated adenocarcinoma, pT1bN0M0. Eleven lymph nodes were harvested; all of which were negative for metastasis. All margins were clear. (b) Moderately differentiated adenocarcinoma, pT3N0M0. Fifteen lymph nodes were harvested, all of which were negative for metastasis. All margins were clear. (c) One case of CBD poorly differentiated adenocarcinoma, pT2N1M0. Eleven lymph nodes were harvested, with one positive for metastasis. All margins were clear. There was one case of low-grade intestinal-type IPMN. Eleven lymph nodes were harvested, all of which were negative for metastasis. All margins were clear.

**Table 4:** Pathology outcomes. IPMN: intraductal papillary mucinous neoplasm; CBD: common bile duct.

Parameter	Data
Pathology	
Pancreatic head ductal adenocarcinoma	3
Duodenal adenocarcinoma	2
CBD adenocarcinoma	1
IPMN (low grade)	1
Harvested lymph nodes	12 (range, 7−19)
Largest tumor size, mean, cm	2.7 (range, 1.5−5)

## Discussion

Pancreatic surgery is widely recognized as one of the most technically demanding procedures in abdominal surgery and is consistently associated with the highest rates of postoperative complications [11,12]. The feasibility of MPRPD has been demonstrated in several studies. Watkins and colleagues reported on outcomes from the first MPRPDs performed across five centers between 2008 and 2014 [13]. Their findings demonstrated that RPD, a significant advancement in modern surgical innovation, can be safely and effectively performed in carefully selected patients receiving care at high-volume medical centers. In a cohort of 92 patients (mean age, 65 ± 12 years), the median operative time was 504 minutes, and the median estimated blood loss was 242 mL. Notably, conversion to open surgery was required in 12 patients (13%). CR-POPF occurred in nine patients (9.9%), including four cases of grade B and five cases of grade C. The rate of severe postoperative complications was 24%, with two (2.2%) postoperative deaths and 10 (10.9%) reoperations. Resection with negative margin was achieved in 75% of patients, and the mean lymph node harvest was 16 ± 8. [13].

Compared with these initial MPRPD experiences, our early results with SPRPD using the da Vinci SP system are encouraging. In our series of seven consecutive cases, the average operative time was 584 minutes, and the mean estimated blood loss was 143 mL. No patient required conversion to either laparoscopic or open surgery. CR-POPF (grade B only) occurred in one patient (14.3%). Negative resection margins were achieved in 85.7% of cases, and the mean number of harvested lymph nodes was 13 ± 6. These findings suggest that the initial outcomes of SPRPD are comparable with those reported for MPRPD and support the safety and feasibility of this approach, despite the small sample size.

Based on our experience with GI and HBP procedures using the da Vinci SP platform, several distinct advantages have been observed. First, the system markedly reduces docking time compared with earlier robotic devices, requiring only a simplified single-docking step. Second, robotic arm collisions are minimized due to the innovative design, which allows three multi-jointed instruments to be introduced through a single arm, aligned and visualized in real time, thereby enhancing both precision and control. Third, the platform incorporates a novel multi-camera mode with cobra-like positioning, providing superior intraoperative visualization. Traditional single-incision approaches to pancreaticoduodenectomy are technically challenging owing to the complexity of the procedure. In SPRPD, a 5-cm left paraumbilical incision is created for insertion of the SP access port. Owing to its central location within the peritoneal cavity, this approach provides a panoramic view of all four abdominal quadrants. Furthermore, the SP system facilitates management of the entire operative field as a unified entity, enabling efficient execution of complex multi-quadrant procedures. In our series, only the first case required one assistant port, whereas the subsequent six procedures were performed as pure SPRPD. Cosmetic outcomes were highly satisfactory, with postoperative scars becoming nearly imperceptible within a few months. Fourth, RPD remains one of the most complex minimally invasive procedures, requiring advanced skill in both laparoscopic and robotic surgery. Challenging steps, such as uncinate process dissection, demand substantial experience. The SP system demonstrated the capability to perform all types of anastomoses, including pancreatojejunostomy, pancreaticojejunostomy with the Blumgart technique, and PG.

Nevertheless, some limitations remain. The current SP system lacks an integrated suction device, stapling system, and energy devices or vessel sealers with endowrist articulation. Further refinements are necessary to address these limitations. Several studies have evaluated the learning curve of RPD. Reports indicate that a single surgeon may require 20−40 cases to achieve initial proficiency [14-18]. More recent analyses from high-volume centers suggest that true technical mastery may require approximately 250 procedures [19,20]. Beyond this threshold, outcomes improved substantially [21]. The largest analysis, comprising 500 cases, demonstrated that improvements continued beyond the initial steep learning curve: intraoperative blood loss and conversion rates decreased after 20 cases, while pancreatic fistula rates declined after 40 cases. The CR-POPF incidence in the final 100 RPDs was 3.3%. Mean operative time decreased to 415 minutes after 80 cases, plateaued at 391 minutes after 240 cases, and averaged 373 ± 76 minutes in the final 100 procedures. After approximately 240 cases, no additional statistically significant reduction in operative duration was observed, suggesting a plateau in surgical efficiency.

In summary, the learning curve for MPRPD is estimated at 20−40 cases for a single surgeon to achieve basic proficiency, while true mastery is generally attained after approximately 250 procedures. Furthermore, it has been suggested that approximately 35 cases are necessary to overcome the learning curve for MPRPD involving vascular resection.

## Conclusions

SPRPD is technically feasible and associated with favorable short-term outcomes, supporting its potential clinical applicability. This approach enables precise anatomical dissection with negative margins and allows for the performance of various types of anastomoses. However, the role of SPRPD as a standard approach remains controversial. Further investigations, including long-term outcomes and randomized controlled trials, are necessary to establish its true clinical value. In addition, the development and adoption of standardized procedural techniques may help shorten the learning curve.
